# Teixobactin Provides Protection against Inhalation Anthrax in the Rabbit Model

**DOI:** 10.3390/pathogens9090773

**Published:** 2020-09-22

**Authors:** William S. Lawrence, Jennifer E. Peel, Satheesh K. Sivasubramani, Wallace B. Baze, Elbert B. Whorton, David W. C. Beasley, Jason E. Comer, Dallas E. Hughes, Losee L. Ling, Johnny W. Peterson

**Affiliations:** 1 Department of Microbiology & Immunology, University of Texas Medical Branch, Galveston, TX 77555, USA; jepeel@utmb.edu (J.E.P.); dwbeasle@utmb.edu (D.W.C.B.); jscomer@utmb.edu (J.E.C.); jpeterso@utmb.edu (J.W.P.); 2 Institute for Human Infections & Immunity, University of Texas Medical Branch, Galveston, TX 77555, USA; ewhorton@gmail.com; 3 Directorate of Environmental Health Effects Laboratory, Naval Medical Research Unit, Dayton, OH 45433, USA; satheesh.sivasubramani.ctr@us.af.mil; 4 Department of Comparative Medicine, University of Texas MD Anderson Cancer Center, Bastrop, TX 78602, USA; wbaze@mdanderson.org; 5 Institutional Office of Regulated Nonclinical Studies, University of Texas Medical Branch, Galveston, TX 77555, USA; 6 NovoBiotic Pharmaceuticals, LLC, Cambridge, MA 02138, USA; dhughes@novobiotic.com (D.E.H.); lling@novobiotic.com (L.L.L.)

**Keywords:** *Bacillus anthracis*, anthrax, inhalation, infection, antibiotic, rabbit model, protective antigen

## Abstract

The use of antibiotics is a vital means of treating infections caused by the bacteria *Bacillus (B.) anthracis*. Importantly, with the potential future use of multidrug-resistant strains of *B. anthracis* as bioweapons, new antibiotics are needed as alternative therapeutics. In this blinded study, we assessed the protective efficacy of teixobactin, a recently discovered antibiotic, against inhalation anthrax infection in the adult rabbit model. New Zealand White rabbits were infected with a lethal dose of *B. anthracis* Ames spores via the inhalation route, and blood samples were collected at various times to assess antigenemia, bacteremia, tissue bacterial load, and antibody production. Treatments were administered upon detection of *B. anthracis* protective antigen in the animals’ sera. For comparison, a fully protective dose of levofloxacin was used as a positive control. Rabbits treated with teixobactin showed 100% survival following infection, and the bacteremia was completely resolved by 24–48 h post-treatment. In addition, the bacterial/spore loads in tissues of the animals treated with teixobactin were either zero or dramatically less relative to that of the negative control animals. Moreover, microscopic evaluation of the tissues revealed decreased pathology following treatment with teixobactin. Overall, these results show that teixobactin was protective against inhalation anthrax infection in the rabbit model, and they indicate the potential of teixobactin as a therapeutic for the disease.

## 1. Introduction

*Bacillus (B.) anthracis*, the Gram-positive bacteria responsible for anthrax infection, has been a focus of the biodefense community for many years because of its past and potentially future use as a bioweapon [1,2]. Inhalation anthrax, which is one of four forms of the disease (the others are cutaneous, gastrointestinal, and injectional), is initiated upon inhalation of anthrax spores into the lungs [3]. While in alveolar spaces, the spores are phagocytosed by macrophages and dendritic cells that then migrate to regional lymph nodes. In the lymph nodes, the spores germinate, and the resultant bacteria multiply and eventually disseminate into the lymphatics, after which the bacteria enter the bloodstream (bacteremia). From circulation, the bacteria are capable of infecting various organs causing significant, widespread tissue pathology [3]. This ultimately leads to death of the host. In the germinated form, *B. anthracis* secrete a tripartite toxin, comprised of protective antigen (PA), lethal factor (LF), and edema factor (EF), which form the active toxins known as lethal toxin (LeTx) and edema toxin (EdTx) [4,5,6]. These toxins have been shown to bring about numerous pathological/pathophysiological effects due to their enzymatic abilities [6,7,8]. In recent years, there has been a renewed interest in this bacterial agent due to the growing concern for the development of multidrug-resistant (MDR) *B. anthracis* strains that could be used as bioweapons. While various antibiotics are currently effective against *B. anthracis* [3,9,10], some of which are included in the Strategic National Stockpile (SNS), these antibiotics could be rendered futile if the bacteria were genetically altered to become resistant [11]. Consequently, there is a continual need to discover and/or develop new therapeutics to combat this potential threat.

Teixobactin is a recently discovered antibiotic produced by a species of β-proteobacteria named *Eleftheria terrae*, which belongs to a Aquabacteria-related genus [12,13]. This organism was discovered using the iChip, a multichannel device that can simultaneously isolate and grow uncultured bacteria [12,14,15]. Studies show that teixobactin, which is an unusual depsipeptide, has tremendous activity against Gram-positive bacteria, specifically by inhibiting cell wall synthesis. It accomplishes this by binding a highly conserved motif of lipid II and lipid III, which are precursors of peptidoglycan and cell wall teichoic acid, respectively [12,16,17]. Thus far, teixobactin has shown efficacy against Methicillin-resistant *Staphylococcus aureus* (MRSA), Methicillin-susceptible *Staphylococcus aureus* (MSSA), Vancomycin Intermediate *Staphylococcus aureus* (VISA), Vancomycin-resistant *enterococci* (VRE), *Clostridium difficile*, *Streptococcus pneumoniae*, *Mycobacterium tuberculosis*, and *B. anthracis*, either *in vitro* or *in vivo*. Astonishingly, the minimum inhibitory concentrations (MIC) for *C. difficile* and *B. anthracis* are as low as 5 and 20 ng/mL, respectively [12,18,19].

The targets of teixobactin are lipid II and lipid III. Lipid II, a peptidoglycan precursor, is composed of one bactoprenol hydrocarbon chain, a disaccharide of N-acetylglucosamine (GlcNAc) and N-acetylmuramic acid (MurNAc), a penta-peptide attached to the MurNAc, and a pyrophosphate group [20]. During bacterial cell wall synthesis, lipid II translocates across the cell membrane to deliver and incorporate its disaccharide and penta-peptide into the peptidoglycan network. In binding the pyrophosphate moiety and the first sugar moiety of lipid II, teixobactin inhibits the incorporation of the disaccharide-pentapeptide into the cell wall, which is a critical step in the cell wall synthesis pathway [16]. The other target of teixobactin is lipid III, a cell wall teichoic acid (WTA) precursor. WTAs are glycopolymers covalently attached to peptidoglycan via linkage to N-acetyl muramic acid sugars, and they account for as much as 60% of the cell wall mass in Gram-positive bacteria. WTAs play a variety of roles including cell shape determination, regulation of cell division, development of antibiotic resistance, and other fundamental facets of Gram-positive bacterial physiology [21,22,23]. When teixobactin binds lipid III, it blocks WTA biosynthesis which causes both the accumulation of toxic intermediates that are lethal to the bacteria as well as the liberation of autolysins that break down the peptidoglycan matrix [12].

As stated previously, the potential development and use of MDR *B. anthracis* strains for nefarious use is a matter of concern [24,25,26,27]. By introducing mutations into crucial bacterial proteins that are targets for current antibiotics, one could render the antibiotics ineffective. However, in the case of teixobactin, the targets are not proteins, but are highly conserved structural components of bacteria [12,16,17]. This suggests resistance through genetic modification of the targets would be very difficult to develop. In fact, resistance to teixobactin could not be obtained in strains of *S. aureus* and *M. tuberculosis* even when plating the bacteria on media with low concentrations of the antibiotic or serial passaging in subinhibitory levels of the antibiotic [12]. Moreover, teixobactin was not toxic against mammalian NIH/3T3 and HepG2 cells at the highest concentration tested of 100 µg/mL [12].

In this blinded study, we evaluated the protection afforded by teixobactin against inhalation anthrax infection in the rabbit model. To our knowledge, this is the first reported work showing the therapeutic potential of teixobactin against inhalation anthrax in an animal model.

## 2. Results

### 2.1. Aerosol Infection Parameters

The average infectious dose of *B. anthracis* Ames spores was 3.29 × 10^7^ (±5.60 × 10^6^) colony forming units (cfu) which corresponds to 329 LD_50_ (50% lethal dose). The duration of aerosol infection, which varied among the animals since each animal was infected individually using real-time plethysmography, was approximately 19 minutes on average. Lastly, the average aerosol spray factor, which indicates aerosol efficiency of the spores, was 2.97 × 10^−6^.

### 2.2. Detection of Protective Antigen

Blood samples were collected from each animal every 6 h beginning 12 h post-infection for detection of PA in serum. Upon detection of PA, the appropriate treatment was initiated for each animal. Among the treatment groups, PA was first detected as early as 12 h post-infection and as late as 36 h post-infection (Table 1), but the majority of the animals were antigenemic by 18 to 24 h post-infection. The concentration of PA in the sera ranged from approximately 0.1 to 6.0 ng/mL with an average of 0.9 ng/mL.

### 2.3. Survival Rate

**Figure 1:**
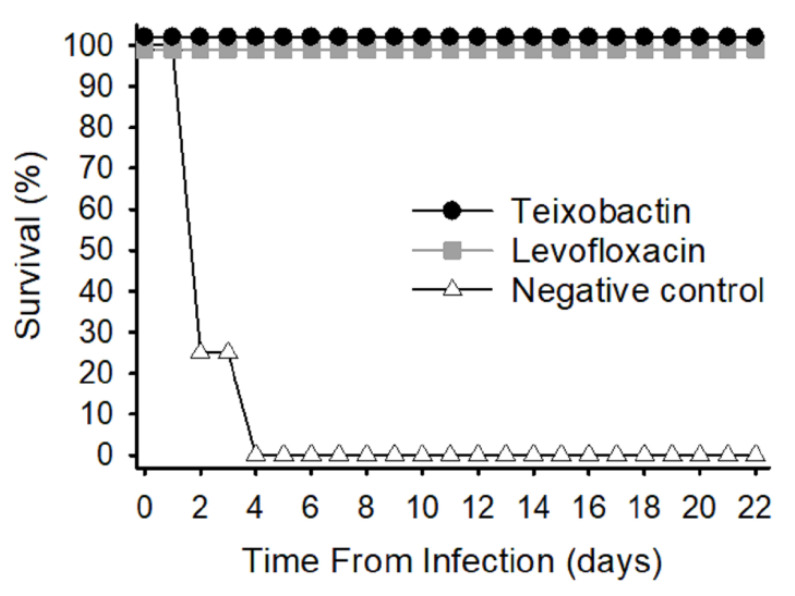
Survival rates of treatment groups infected with 329 LD_50_ (50% lethal dose) anthrax spores. Three groups of animals were infected by aerosol with approximately 3.29 × 10^7^ colony forming units (cfu) *Bacillus (B.) anthracis* Ames spores. Treatments, administered intravenously, were initiated upon detection of protective antigen (PA), and animals were monitored for 21 days post-treatment initiation.

Survival was monitored for 21 days post-treatment initiation (Figure 1). The group receiving teixobactin at 5 mg/Kg intravenously (IV), once a day (SID) for 5 days exhibited 100% survival against the lethal infectious dose of anthrax spores, and the survival percentage was significantly higher than that of the negative control group (*p*-value < 0.05) Likewise, the positive control group that received levofloxacin (12.5 mg/Kg IV, SID for 5 days) was also fully protected. The levofloxacin dose selected for this study was equivalent to a humanized dose. The negative control group that received only diluent showed no survival. These results show that teixobactin was protective against inhalation anthrax infection in this animal model.

### 2.4. Temperature Response

**Figure 2:**
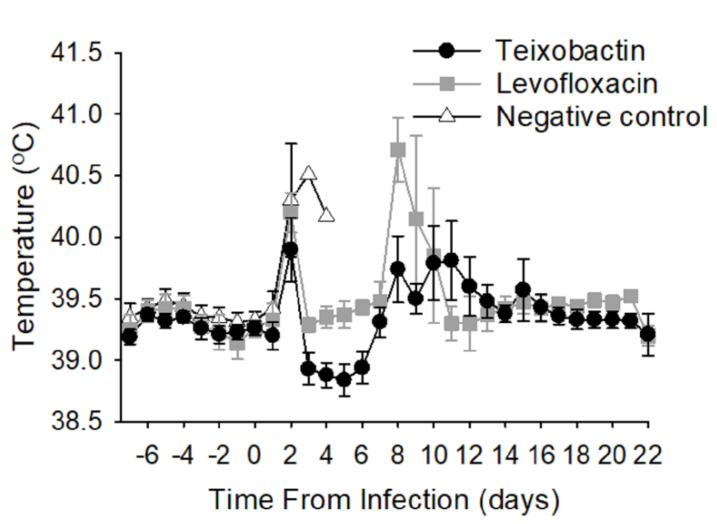
Average temperature responses of treatment groups during infection. Animals were surgically implanted with temperature data loggers prior to the study. Temperatures were recorded every 10 seconds before and after infection, and the results are presented as 24-hour moving averages. Each time-point shows the group averages with standard error bars.

All treatment groups exhibited febrile responses due to the bacterial infection approximately 2 days post-infection (time of infection at Day 0) (Figure 2). These initial temperature elevations subsided in the teixobactin and levofloxacin groups; however, the temperature remained elevated in the negative control group until Day 4 post-infection after which time all the animals in the negative control group had been euthanized. The teixobactin-treated group exhibited a transient decline in temperature 3 to 6 days post-infection that was not seen in the levofloxacin-treated group. Both antibiotic-treated groups had a secondary febrile response within 8 to 12 days post-infection, the magnitude of which appeared greater for the levofloxacin-treated group and less for the teixobactin group; however, differences in temperature among these groups during this time-period (8 to 12 days post-infection) were not significant. Ultimately, the temperatures returned to baseline for both the teixobactin and levofloxacin groups. In view of the fact that fever is a common response to anthrax infection in animal models, these results demonstrate that teixobactin was effective at treating the infection.

### 2.5. Level of Bacteremia

The level of bacteremia was measured in whole blood at multiple time-points post-infection and post-treatment initiation; however, only certain time-points are presented in Table 2 since the values for the remaining time-points were either zero or not determined (due to euthanasia of the animals). Terminal samples were also collected either at the end of the post-infection period (for surviving animals) or at the time of euthanasia (for animals that we euthanized due to their clinical condition). Approximately half the animals treated with teixobactin were bacteremic by 24 h post-infection; however, by 24 h post-treatment initiation, only 1 out the 7 animals treated with teixobactin was bacteremic. By 48 h post-treatment initiation, all teixobactin-treated animals were abacteremic, and they remained so for the remainder of the post-infection period. Similarly, the infection was resolved early following treatment with levofloxacin. Half the negative-control animals were bacteremic by 24 h post-infection, and all were bacteremic by 24 h post-treatment initiation (given diluent). These animals remained bacteremic up to the time of euthanasia. These results show that teixobactin was capable of rapidly clearing *B. anthracis* from the circulating blood.

### 2.6. Bacterial Load in Tissues

**Figure 3:**
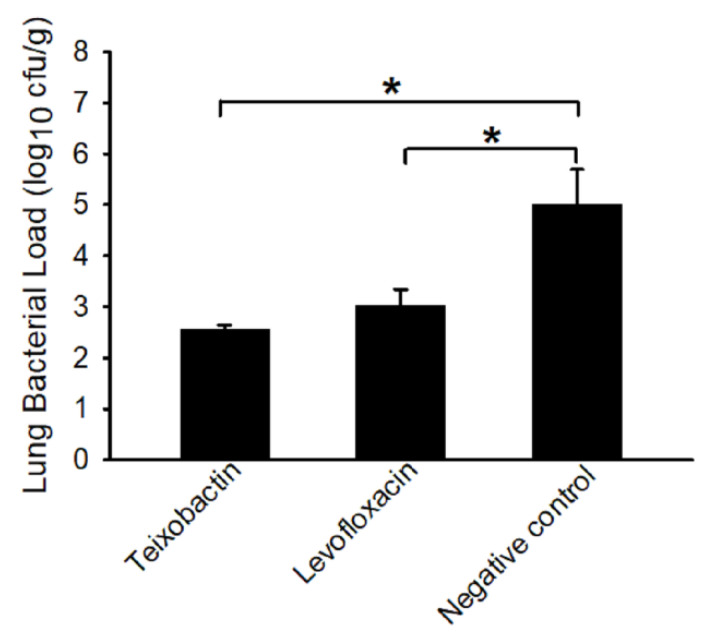
*B. anthracis* load (log_10_) in lung tissues collected at termination. Tissues were collected after euthanasia of the animals and homogenized in sterile water. The homogenates were plated onto trypticase soy agar plates containing 5% sterile sheep blood (TSAB) plates and incubated at 37 °C for 16–24 h. Colonies from the plates were then enumerated. The bacterial loads are presented as the average log_10_ cfu per gram of tissue (with standard error bars). The asterisk denotes significance (*p*-value < 0.05).

In the case of the teixobactin and levofloxacin-treated animals, the bacteria were detected only in the lungs. The negative control animals, on the other hand, possessed bacteria in the brain, mediastinal lymph nodes, and spleen in addition to the lungs (Table 3). Importantly, the bacterial load in the lung tissue of the antibiotic-treated (teixobactin or levofloxacin) animals was significantly (*p*-value < 0.05) less than that of the negative control animals (Figure 3). This demonstrates that teixobactin was able to clear *B. anthracis* from various tissues and limit progression of the infection in the lungs.

### 2.7. Anti-PA Antibody Response

**Figure 4:**
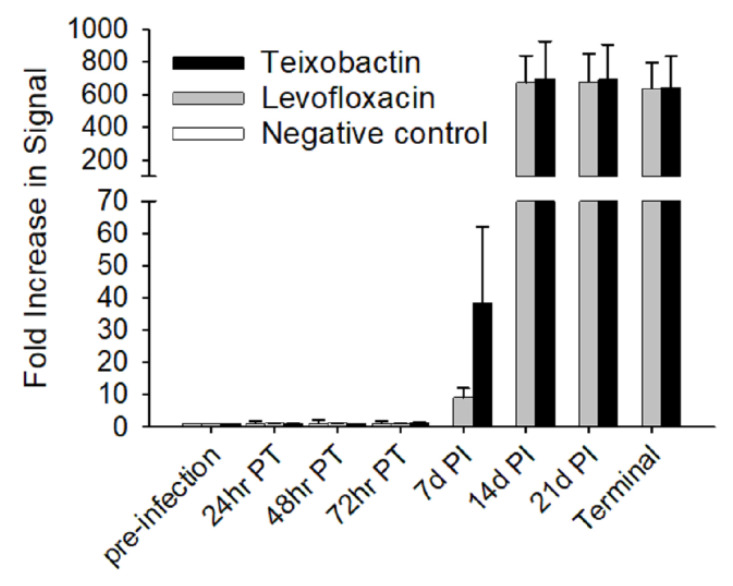
Anti-PA antibody responses of treatment groups during anthrax infection. Sera samples were diluted 10^−3^ and plated onto PA-coated plates. Detection was accomplished using SULFO-TAG labeled anti-rabbit antibody. The responses were measured via electrochemiluminescence. To determine the fold increase in signal, the respective pre-infection time-point for each treatment group was used as the reference. Each time-point shows the group averages pre-infection, post-treatment initiation (PT) and post-infection (PI) with the standard error bars.

By 14 days post-infection, the animals treated with teixobactin and levofloxacin exhibited dramatic rises in the amount of serum anti-PA antibodies, and the antibody levels remained steady for the remaining time-points (Figure 4). There were no significant differences in serum anti-PA antibody titers between the two antibiotic-treated groups; however, both antibiotic-treated groups had a significant (*p*-value < 0.05) increase over time. As expected, the negative control group showed no increase in anti-PA antibody levels over time since they succumbed to the anthrax infection before a humoral response could be mounted. These results show that teixobactin provided protection prior to the production of protective anti-PA antibodies.

### 2.8. Necropsy and Histopathology

At necropsy, the animals in the negative control group consistently showed enlarged, hemorrhagic mediastinal lymph nodes and pulmonary congestion. Also evident was the portal pattern in the liver. The teixobactin-treated animals, on the other hand, exhibited no remarkable findings similar the levofloxacin-treated animals.

**Figure 5:**
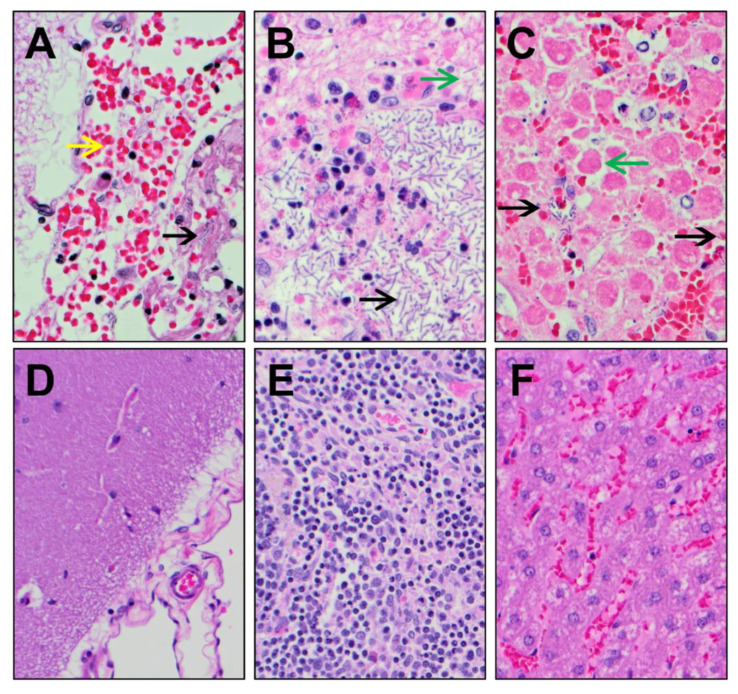
Histopathological images of tissues collected from negative control and teixobactin-treated animals. Tissues sections were routinely processed, embedded in paraffin, sectioned at 5 µm, and stained with hematoxylin and eosin (H & E), and then evaluated by light microscopy. (**A**) Brain, (**B**) mediastinal lymph node, and (**C**) liver tissues collected from negative control animals (at 400× magnification) showed the presence of *B. anthracis* rods (black arrow), hemorrhage (yellow arrow), and/or necrosis (green arrows). The (**D**) brain, (**E**) mediastinal lymph node, and (**F**) liver tissues collected from teixobactin-treated animals (at 200× magnification) showed minimal pathology with no *B. anthracis* rods.

Histopathological analysis revealed in the negative control animals the presence of lesions that were consistent with anthrax and included cellular/tissue degeneration/necrosis, congestion, hemorrhage, edema, inflammatory infiltrates (fibrinous, mixed cellular), and/or the presence of bacteria in multiple tissues (particularly the brain, mediastinal lymph nodes, and liver) (Figure 5). The severity of the lesions varied from 1 to 4 of 4. Conversely, the animals treated with teixobactin had lesions that were generally mild relative to the animals in the negative control group, with the severity varying from 1 to 2 of 4 (Figure 5). Lastly, lesions from the animals treated with levofloxacin were nonspecific and/or similar to animals in the teixobactin-treated group, and the severity was 1 to 2 of 4 (not shown). Altogether, these results show that teixobactin treatment reduced the tissue pathology associated with anthrax infection.

## 3. Discussion

The United States Food and Drug Administration (FDA)-approved antibiotics used for post-exposure prophylaxis (PEP) following exposure to aerosolized anthrax spores are doxycycline, ciprofloxacin, levofloxacin, and parental procaine penicillin G [28]. The Centers for Disease Control and Prevention (CDC) recommends a 60-day regimen of doxycycline or ciprofloxacin along with a three-dose series of anthrax vaccine adsorbed (AVA), the current FDA-approved anthrax vaccine. Importantly, undisclosed quantities of these antibiotics are included in the SNS for the emergency health security of the United States and its territories. Even so, while currently there are antibiotics effective against anthrax infection, there remains a need for novel antibiotics capable of combating MDR *B. anthracis* strains that would render previously effective drugs futile. In 2009, The United States Department of Homeland Security (DHS) identified multi-drug resistant (MDR) anthrax as a Chemical, Biological, Radiological, Nuclear (CBRN) agent that poses a material threat to national security [29]. Therefore, the necessity for new antibiotics, those that would be broad-spectrum and would retain their potency after decades of use, is evident.

Teixobactin, a recently discovered antibiotic effective against Gram-positive bacteria, is one such candidate antibiotic that would be effective against not only antibiotic-sensitive bacteria but also MDR bacteria. The mechanism of action of teixobactin binding highly conserved non-protein structural motifs in Gram-positive bacteria suggests it is resilient to antibiotic-resistance [12,16,17]. In this study, teixobactin was protective against inhalation anthrax infection using New Zealand White rabbits infected with approximately 300 LD_50_ *B. anthracis* Ames spores. Importantly, teixobactin treatment was not initiated until infection was confirmed by detection of the *B. anthracis* PA in the animals’ blood (trigger-to-treat). Studies such as this are quite advantageous, particularly in the case of therapeutic testing against anthrax infection, since clinically, the disease initially presents with nonspecific symptoms that do not allow for a definitive clinical diagnosis.

The results of this study show that teixobactin was also capable of removing *B. anthracis* from both the circulation and all but one of the collected tissues. *B. anthracis* were, however, detected in the lungs of all animals at termination, even in the animals that survived the entire post-infection period, albeit in a lesser amount relative to the untreated control animals. Anthrax spores persist in the lungs for extended periods of time. In fact, dormant spores were recovered from nonhuman primates and mice weeks or months post-exposure [30,31,32]. Delayed onset of anthrax infection is also attributed to dormant spores [33], presumably due to asynchronous germination of the spores. This may be the reason for the secondary fever response observed among the antibiotic-treated groups in this study. Because of anthrax spores’ ability to persist in lung tissue for long periods of time, the CDC-recommended duration of PEP antimicrobial therapy is 60 days [34].

Bacterial clearance of the tissues may have been partly due to the animals’ own acquired immunity since tissues of the surviving animals were harvested approximately 22 days post-infection, and a robust anti-PA antibody response was already evident by 14 days post-infection. In our experience with the NHP and rabbit models of anthrax infection, we find that if a therapeutic can protect the animal for 7–14 days post-infection, survival is likely even if therapy is discontinued prior to 7 days post-infection. This most likely occurs because the animals are given adequate time for antibody development/maturation. In this study, we showed that teixobactin was capable of protecting the animals during the peak time of infection (2–5 days post-infection) which occurs prior to the development of protective antibodies. This ability is crucial for any prospective therapeutic used to treat anthrax infection.

Teixobactin was able to clear the infection as evidenced by the temperature response of the teixobactin-treated animals; however, these animals also exhibited a reduction in temperature during treatment. This could possibly have been a mild side effect of the drug. Extended use of antibiotics in hindgut fermenters such as rabbits is known to cause antibiotic-associated enterotoxemia, a condition in which antibiotic use disrupts the delicately-balanced intestinal flora (dysbiosis) thereby causing gastrointestinal stasis and overgrowth of opportunistic organisms such as *Escherichia coli* and *Clostridium* spp. [35,36]. Hypothermia is a sign of this condition. Nonetheless, this effect of the drug may be exclusive to rabbits and other herbivores due to their extremely sensitive digestive systems.

In this study, teixobactin, administered at a concentration of 5 mg/Kg SID for 5 days upon detection of PA, was protective against inhalation anthrax infection. For future studies, we intend to test the level of protection provided by teixobactin at lower concentrations and at later administration times.

## 4. Materials and Methods 

### 4.1. Bacteria

*B. anthracis* Ames spores were grown in modified Schaeffer’s medium using a computer-controlled New Brunswick fermenter. After inoculation, the fermenter is operated with aeration at pH 7.0–7.5 with pH control for approximately 4 days, after which time the crude spore content of the culture is aseptically harvested by centrifugation and washed with sterile molecular grade water. The spores are purified by density gradient centrifugation using sterile MD-76. Visual observation of the spores at 400 × by phase-contrast microscopy during each step of purification is performed to ensure production of a homogeneous suspension of highly refractile spores. The purified spores were aliquoted and stored at 4 °C in sterile molecular grade water containing 1% phenol and 0.05% Tween 20. The inventory of spores is periodically monitored by the UTMB Office of Environmental Health and Safety as well as the CDC.

### 4.2. Animal Experiments

This study was conducted as part of an NIAID-funded contract (Contract No. HHSN272201700040I/ HHSN2720008 PCMID Task Order A12), and the study design, which includes the number of animals, was determined prior to receiving the contract. All animal procedures conducted for this study were approved by the University of Texas Medical Branch Institutional Animal Care and Use Committee (IACUC). New Zealand White specific-pathogen-free rabbits (3.0–3.5 kgs) were obtained from Envigo (Denver, PA, USA). A venous access port (VAP) was surgically implanted into the external jugular vein of each animal by Envigo to facilitate collection of multiple blood specimens and administration of treatments. Each animal was also implanted intraperitoneally with a DST micro-T temperature data logger (Star-Oddi Ltd, Gardabaer, Iceland) for the purpose of recording the animals’ temperatures for the entire in-life period.

While anesthetized (using ketamine/xylazine), the animals were infected by aerosol with an approximate dose of 300 LD_50_ of purified *B. anthracis* Ames NR3838 spores (3.0 × 10^7^ cfu) using a Biaera aerosol control platform (Biaera Technologies LLC, Hagerstown, MD, USA) fitted with a muzzle-only aerosol chamber using computer control of humidity, pressure, and airflow. Real-time plethysmography (Data Sciences International, St. Paul, MN, USA) was performed on each rabbit during infection. We used a 6-jet Collison nebulizer to generate the aerosol. Aerosol samples were collected continuously using an all-glass aerosol Bio-Sampler (SKC, Inc, Eighty Four, PA, USA) during each exposure for confirming the infectious dose of spores for each animal by serial dilution and plating on blood agar plates. The duration of aerosol delivery was based on the respiration rate of each animal and the total volume of inspired air monitored by the Biaera aerosol system computer.

Blood specimens were collected from each animal, via the VAP, prior to infection and at specified times post-treatment and post-infection. The blood samples were used to perform the necessary assays. Blood specimens were collected from the central ear artery instead of the VAP starting from day 7 post-infection because *B. anthracis* in the blood of bacteremic animals have the potential to colonize the VAP, thereby possibly contaminating successive samples drawn from the port. Starting immediately after a positive serum PA titer post-infection (trigger-to-treat), animals were given either 5 mg/Kg teixobactin SID for 5 days (*n* = 7), 12.5 mg/Kg levofloxacin SID for 5 days (*n* = 2), or diluent SID for 5 days (*n* = 4), IV via the implanted VAP, once a day for 5 days. Teixobactin, obtained from NovoBiotic Pharmaceuticals, LLC. (Cambridge, MA, USA), was isolated as described [12]. Briefly, the cell biomass of culture fermentation was first extracted with aqueous acetonitrile followed by extraction with n-butanol (n-BuOH). The n-BuOH extract was then subjected to preparatory high-performance liquid chromatography (HPLC) (SP:C18, MP:H_2_O/MeCN/0.1% TFA). The resulting trifluoroacetic acid (TFA) salt was converted to a hydrochloride salt using a Dowex (134 Cl_2_ form) column. Endotoxin was removed by filtration through a Pall 3K MW Centrifugal filter, and the solution lyophilized to leave a white powder. Prior to treatment administration, teixobactin was solubilized in 5 mg/mL 1,2-distearoyl-sn-glycero-3-phosphoethanolamine-N-[methoxy(polyethylene glycol)-2000] (ammonium salt) (Avanti Polar Lipids, Inc, Alabaster, AL, USA) with 5% dextrose (Sigma-Aldrich, Inc, St. Louis, MO, USA). This protection study was conducted in a blinded fashion; therefore, personnel administering treatments were not privy to the animals’ treatment designations.

After infection, animals were observed four times daily for signs of disease progression. Any rabbits exhibiting respiratory distress and/or immobility were euthanized with an intravenous administration of a commercial euthanasia solution (Fatal-Plus, Vortech Pharmaceuticals LTD, Dearborn, MI, USA). Like treatment administration, personnel performing clinical observations were blinded to group assignment.

### 4.3. Protective Antigen Detection

*B. anthracis* PA was measured in serum using a rapid PA-ECL screening assay (MesoScale Discovery, Gaithersburg, MD, USA). To quantitate the levels of PA in each serum sample, a standard curve (0–100 ng/mL) was analyzed in parallel on each assay day. Test samples were assayed in duplicate. The concentration of each test sample was extrapolated from the standard curve.

### 4.4. Assessment of Bacteremia and Bacterial Load

Bacterial concentration in the blood was determined using an automatic serial diluter and plater (easySpiral Dilute; Interscience Laboratories, Inc, Woburn, MA, USA). Whole blood, diluted in sterile water, was plated onto trypticase soy agar plates containing 5% sterile sheep blood (TSAB) and incubated at 37 °C for 16–24 h. Colonies from the plates were then enumerated using an automatic colony counter (Scan 500; Interscience Laboratories). Bacterial colonies having morphology typical of *B. anthracis* were subcultured and confirmed as *B. anthracis* with bacteriophage γ.

Bacterial/spore load was also determined in lung, lymph node (mediastinal), brain, and spleen. These tissues were homogenized in PBS with 1% peptone using a Stomacher 80 MicroBiomaster homogenizer (Seward Ltd, Bohemia, NY, USA), and the homogenate was serially diluted in sterile water and plated onto TSAB plates using the automatic diluter/plater and incubated at 37 °C for 16–24 h. Colonies from the plates were then enumerated using an automatic colony counter (Scan 500; Interscience Laboratories), and the bacterial load was presented as cfu per gram of tissue. Bacterial colonies having morphology typical of *B. anthracis* were subcultured and confirmed as *B. anthracis* with bacteriophage γ.

### 4.5. Quantitation of Serum Anti-PA Antibody

Anti-PA antibodies were measured in rabbit sera via electrochemiluminescence similar to the PA-ECL screening assay. Biotinylated recombinant PA83 (List Biological Laboratories, Inc, Campbell, CA, USA) was bound to streptavidin-coated plates (MesoScale Discovery, Gaithersburg, MD, USA) and used as the capture antigen. Detection was accomplished using SULFO-TAG labeled anti-rabbit antibody and read buffer (MesoScale Discovery). Sera were diluted 10^−3^ prior to measuring in order to have all samples within the limits of detection. To determine the fold increase in signal, the respective pre-infection time-point for each treatment group was used as the reference.

### 4.6. Necropsy and Histopathology

Following euthanasia of the animals, gross necropsy was performed on all animals. The collected tissues (lung, mediastinal lymph nodes, brain, and spleen) were perfused with 10% phosphate buffered formalin. Tissues sections were routinely processed, embedded in paraffin, sectioned at 5 µm, and stained with hematoxylin and eosin (H&E) and evaluated by light microscopy. A four-level severity scale was used when appropriate, utilizing the following terms: minimal (1 of 4), mild (2 of 4), moderate (3 of 4), and marked (4 of 4).

### 4.7. Statistics

Statistical analyses were performed using the statistical package NCSS (NCSS, LLC, Kaysville, UT, USA). Differences between groups were tested using one-way Analysis of Variance (ANOVA) and Tukey–Kramer’s test.

## 5. Conclusions

In conclusion, teixobactin was protective against inhalation anthrax infection in the rabbit model, and consequently, has the potential as an effective therapeutic against the disease.

## 6. Patents

NovoBiotic Pharmaceuticals, LLC owns the patent rights to teixobactin. This includes U.S. patents 9,163,065 (issued 10/20/2015); 9,402,878 (issued 8/2/2016); and 10,414,800 (issued 9/17/2019) as well as U.S. patent applications 16/529,342 (filed 8/1/2019) and 16/969,598 (filed 8/13/2020).

## Figures and Tables

**Table 1:** Detection of protective antigen in rabbit sera.

Treatment Group	Animal Identification Number	Time of PA Detection (Time Post-Infection)	Concentration of PA (ng/mL)
Teixobactin	4490	18 h	0.230
	4493	24 h	1.000
	4497	36 h	6.009
	4498	24 h	0.302
	44873	18 h	0.566
	44881	18 h	1.180
	44882	24 h	0.170
Levofloxacin	4499	24 h	0.312
	44874	18 h	0.419
Negative control	4494	12 h	0.099
	4496	30 h	0.360
	44872	24 h	0.817
	44879	18 h	0.920

PA—protective antigen.

**Table 2:** Bacteremia level.

Treatment Group	Animal No.	Bacteremia Level (cfu/mL)			
		24 h PI	24 h PT *	48 h PT *	Terminal
Teixobactin	4490	0	0	0	0
	4493	1.27 × 10^2^	0	0	0
	4497	0	0	0	0
	4498	0	2.00 × 10^1^	0	0
	44873	5.12 × 10^4^	0	0	0
	44881	5.23 × 10^4^	0	0	0
	44882	0	0	0	0
Levofloxacin	4499	0	0	0	0
	44874	1.74 × 10^4^	0	0	0
Negative control	4494	2.63 × 10^4^	1.03 × 10^6^	euthanized	1.09 × 10^8^
	4496	0	6.90 × 10^2^	2.15 × 10^4^	4.12 × 10^7^
	44872	0	8.00 × 10^3^	euthanized	3.23 × 10^3^^#^
	44879	7.35 × 10^4^	8.57 × 10^6^	euthanized	7.30 × 10^7^

PI—post-infection; PT—post-treatment initiation; cfu—colony forming unit; * Negative control animals were given diluent; # actual terminal bacteremia of animal 44872 could have been hampered by blood clotting or technical problems in acquiring the blood sample.

**Table 3:** Bacterial load in tissues of negative control animals.

Animal No.	Bacterial Load (cfu/g)			
	Lung	Brain	Lymph Node	Spleen
4494	6.96 × 10^4^	3.54 × 10^5^	1.16 × 10^6^	9.35 × 10^6^
4496	7.90 × 10^3^	4.80 × 10^4^	2.56 × 10^7^	1.49 × 10^4^
44872	2.26 × 10^4^	6.83 × 10^6^	2.94 × 10^6^	1.42 × 10^3^
44879	9.40 × 10^6^	5.46 × 10^6^	2.44 × 10^7^	1.84 × 10^6^
